# Tumor Biochemical Heterogeneity and Cancer Radiochemotherapy: Network Breakdown Zone-Model

**DOI:** 10.3390/e24081069

**Published:** 2022-08-02

**Authors:** Argyris Dimou, Panos Argyrakis, Raoul Kopelman

**Affiliations:** 1 Department of Physics and Complexity Center, University of Thessaloniki, 54124 Thessaloniki, Greece; ardimou@physics.auth.gr; 2 Department of Chemistry, and Rogel Cancer Center, University of Michigan Health System, Ann Arbor, MI 48109, USA; kopelman@umich.edu

**Keywords:** hypoxia heterogeneity, tumor radiotherapy, inverse percolation shell model, monte-carlo simulations, oncology radiation modelling, network breakdown

## Abstract

Breakdowns of two-zone random networks of the Erdős–Rényi type are investigated. They are used as mathematical models for understanding the incompleteness of the tumor network breakdown under radiochemotherapy, an incompleteness that may result from a tumor’s physical and/or chemical heterogeneity. Mathematically, having a reduced node removal probability in the network’s inner zone hampers the network’s breakdown. The latter is described quantitatively as a function of reduction in the inner zone’s removal probability, where the network breakdown is described in terms of the largest remaining clusters and their size distributions. The effects on the efficacy of radiochemotherapy due to the tumor micro-environment (TME)’s chemical make-up, and its heterogeneity, are discussed, with the goal of using such TME chemical heterogeneity imaging to inform precision oncology.

## 1. Introduction

This paper extends our previous work [1] that correlated a tumor’s lattice heterogeneities with its radiochemotherapy efficacy, extending it now from an idealized lattice model to a more realistic network model, using a new mathematical approach. It also arrives at some recommendations towards optimized treatment protocols. It is well known that around a tumor there is a special micro-environment (TME) with special characteristics, such as a density gradient, which is very crucial when considering the proper therapies that need to be applied. It is well known that tumors have a center of mass that contains the tumor cells, and this is true in all cases, such as in the original location of the tumor, or at a colony due to metastasis, or even in a laboratory experiment in a xenograft animal model due to implantation. These cells extend throughout the TME in shapes that may have various geometries, some of them very clear or in other cases with a fractal structure. Here we study models that contain such heterogeneities, as developed by the density gradient in the TME. Notably, beyond this mass distribution of the tumor cells, the TME may have a heterogeneity in the distribution of the chemical components, relating to its oxygen content (depletion of O_2_, i.e., hypoxia), acidity (increase in H^+^, i.e., lowering of pH, i.e., acidosis), or extracellular potassium ions (excess of K^+^, i.e., hyperkalemia). Such information has recently become amenable to the novel method of chemical imaging [2]. In order to understand a specific tumor’s biology, we first need to understand the mass and chemical component distribution, the density gradient, and the connectivity network heterogeneity. All these will have important implications for the optimal medical treatment. The drug and the imaging contrast agent need to overcome the difficulty of the tumor penetration, and certainly both the therapy and the imaging will be affected by such difficulty [3]. To make this point, since we know of the TME’s acidity, if one employs chemotherapy, this should be applied only to the tumor periphery, because the TME’s acidity does not allow for the drug to reach the tumor center.

Since we know that the O_2_ concentration is lower at the center of a tumor and higher at its periphery, we clearly see that radiation therapy will not be as effective, and in addition may also affect its combination with chemotherapy. Furthermore, it is also possible that the distribution of hyperkalemia (excess of extracellular K^+^) in the TME may affect the success of immunochemistry [2]. Precision oncology may thus depend on the physical and chemical imaging of the patient’s tumor’s heterogeneities.

In the current work, we build a computational model to mimic the heterogeneities at the point of the network’s break-up, which may now include the TME’s acidity (pH) and its O_2_ concentration. The known therapies today include chemotherapy, radiation therapy, combination therapy, immunotherapy and/or surgery. Therefore, direct knowledge of these heterogeneities could show the way towards the optimal treatment route. For the properties that we investigate here, our model refers to the standard combination therapy, which involves radiation therapy as the first step, followed by chemotherapy.

Our results may also be relevant to the understanding of existing protocols that have been empirically derived [4]. It is well known that the earliest and still most common tumor treatment is via chemotherapy [4], i.e., by employing drugs. However, such drug doses have a severe drawback, as they are limited in their efficiency by their notorious side effects. Additionally, tumors use one of their “chemical weapons”, specifically, the acidity (acidosis) of the TME, and thus, they resist chemotherapy [5,6,7,8,9]. This “acidosis” (low pH) of the TME has been discovered over a century ago by Warburg [10]. Unfortunately, drug molecules decompose in the presence of such acidity. Thus, the right treatment protocol now must start with radiation therapy, which is then followed by chemotherapy [4]. The idea now is, instead of having a uniform TME in the area surrounding the tumor, to initially break-up the extended network of the tumor cells into a number of isolated “clusters”. The drug molecules will be now be able to avoid the acidic portions of the TMEs, due to cluster break-up, and thus they will survive until reaching the tumor cells, to be able to properly operate their function. The topic of this study is to develop a mathematical model of such a break-up of the tumor network, in order to be able to plan the proper therapy.

In our previous recent work, we used a randomized two-component lattice model. Such a well-known model shows a phase transition that depends on the ratio of the concentrations of the two components, the so-called percolation model [11,12]. Such a transition is higher order than one, the customary phase transitions, and it is highly nonlinear, as it is properly described by power laws. Geometrically, it describes the formation or break-up of a connected cluster. The formation of such an extended cluster is mathematically equivalent to its break-up process, with the break-up process being called inverse percolation [13]. Both the network formation and its break-up do occur at a “critical concentration” of the relevant component [11,12]. Here, we extend our previous work on lattices of the break-up of the largest connected cluster, as we now employ random networks of the Erdős–Rényi type [14,15]. We model a real tissue network by such an Erdős–Rényi network that is being broken-up, and thus it contains two parts, live and dead tumor cells, with the dead cells due to radiation treatment. In the new network model, we randomly break links between the nodes and totally remove nodes, in an effort to mimic how cells are randomly killed by the radiation. Additionally, with the presence of hypoxia (the absence of tissue oxygen), tumors may have a “chemical weapon” against radiation therapy. This low concentration of oxygen in the TME has also been known for over a century, also due to Warburg [10]. As the tumor cells depict an accelerated growth and multiplication, resulting in an enhanced metabolism, this leads to hypoxia. Of course, the presence of O_2_ molecules controls the chemical mechanism of cell-kill by radiation. Specifically, the O_2_ molecules have a “triplet” ground state and also a higher energy “singlet” state. The radiation energy moves the molecules from their triplet to their singlet state. The singlet oxygen that is produced kills the cells, and as such it has been called “killer oxygen”, as it produces the so-called “reactive oxygen species” (ROS) [16]. The OH radical molecule is a typical ROS member, while the singlet oxygen molecule itself is another one. The oxygen depletion will be not be uniform in the entire TME, but will be higher at the tumor’s center and lower away from the center at its periphery. This is because in the periphery the metabolized oxygen molecules are constantly replenished by oxygen diffusion from the nearby, oxygen-rich, normal tissue which has zero hypoxia. We thus build a model to include the above attributes where the radiation-based cell-kill may be most effective at the tumor’s outer shell (periphery) and least effective at its center. This leads us to use distinct zones in the Erdős–Rényi network, zones which have different removal probabilities. We thus apply an originally random distribution of tumor cells, with a zone-to-zone density gradient of kill probability. As a first step towards illustrating this approach, we used earlier a simple two-dimensional “onion-like” shelled lattice model [1]. We showed in that work that the “critical concentration”, for the live tumor cell network break-up, does depend strongly on the ratio of removal probabilities in the different zones. We discuss the potential ramifications for radiotherapy and combination radiochemotherapy, with suggestions for an optimally shaped radiation beam and potential methods for tumor oxygenation before radiation. We give graphical illustrations of our preliminary insights regarding the efficacy of the radiotherapy. It is true that more specialized geometries may be needed in the future that are characteristic of specific tumors and their specific TMEs, thus necessitating personalized precision radiation oncology therapy.

In this model, we focus on quantities such as the giant component, but we have to acknowledge similar works that have presented other measures as the number of nodes and edges in the generalized k-core against attacks in the network [17,18]. For the purposes of completeness, we cite [19] where a wide range of mathematical and computational tools for cancer research are analysed.

## 2. Method of Simulation

**Figure 1:**
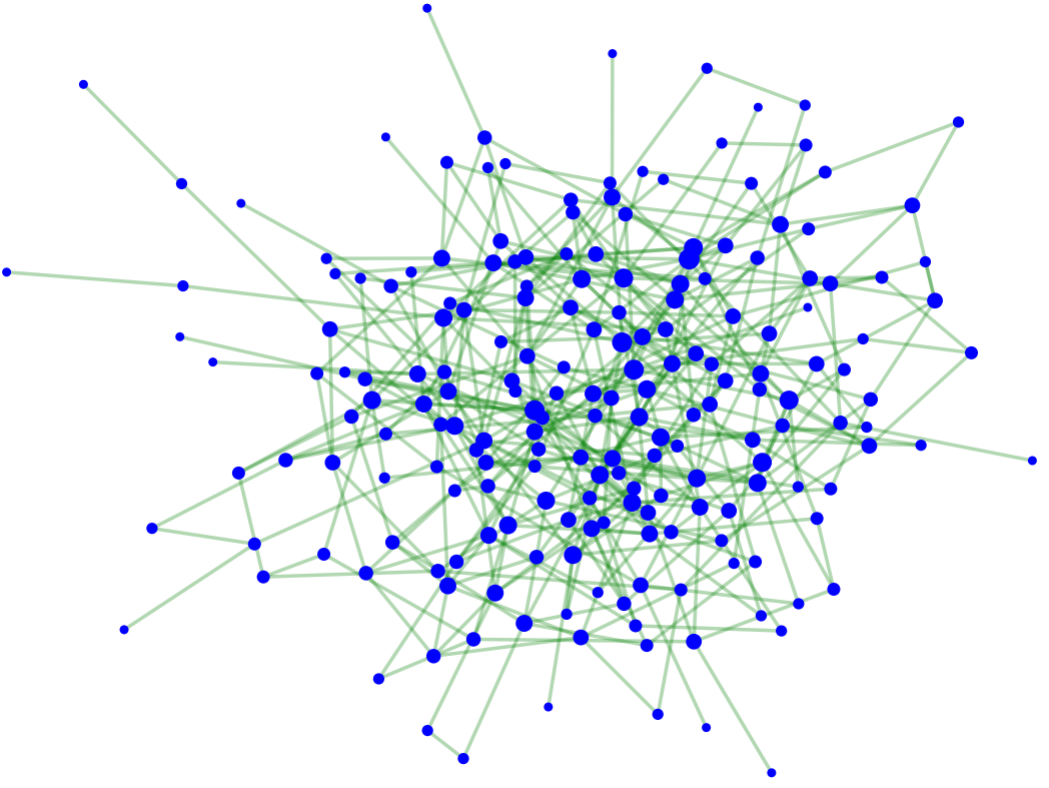
Erdős–Rényi network with N=200 and <k>=4. Nodes with larger *k* appear larger.

We generate random networks of the Erdős–Rényi type with N nodes [20,21]. The Erdős–Rényi type of network, also called random network, is a network type in which any two nodes are connected with a pre-defined probability, p. Thus, when constructed, each node is connected to a number of $k_{i}$ nodes, where $k_{i}$ is the degree of node *i*. A characteristic value of the network is <k>, the average degree of all nodes of the network. The probability distribution of the k nodes results in a Poisson distribution. In Figure 1 we present a typical network of this type. We expect that the nodes with the higher degree would be the most central and the most connected nodes in the network. However, the degree of the nearest neighbors of each node is equally important. As stated in [22], the k-shell of a node reveals how central this node is in the network with respect to its neighbors, meaning that a higher k-value signifies a more central node belonging to a more connected neighborhood in the network.

We construct two (2) models in which the nodes are divided into zones with different removal probabilities. In the first model (k-sorted model), we rank all nodes from highest to lowest according to the degree that they have. Then we generate two zones, one containing all nodes with the highest degree, down to the node with the median degree, and a second zone containing the nodes with the median degree and down to the nodes with the smallest degree. In the second model (k-shell model), we use the well-known k-shell decomposition, thus taking the network apart in trying to get to its core, i.e., the part of the network with the highest connectivity. Once we do this, we again divide the network into two (2) zones. We then start removing nodes randomly with different probabilities in the two zones. When a node is removed, all of its links are also removed. When nodes are picked from the outer zone, they are always removed with probability $p_{ext}=1$. When they are picked from the inner zone, they are removed with a probability equal to $\left(1-r\right)*p_{ext}$ where *r* is defined the same way as in [1]. It is the rate of the reduction of removal probability between the two zones in both models. When r=0 then the removal probability is 1. We vary the value of *r* in the range 0<r<1.

After removing several nodes, we reach the point when there is no longer a spanning cluster in the network, as it has now been broken up into a large number of pieces. The point of this breakdown [23,24] is reached when κ=2, where $\kappa =\frac{<k^{2}>}{<k>}$. When this point is reached, we stop the removal process.

## 3. k-Sorted Model

This is the model with the sorted k values. We divide all the network nodes into 2 zones according to their degree, i.e., the more connected nodes (higher k values) will belong to the internal zone and the less connected ones (lower k values) to the external zone. We calculate the median k of the k values of all the network nodes and assume that this k will be the limit separating the 2 zones. Every node that has a larger k will belong to the internal zone, while every node with smaller k belongs to the external zone. In order to divide the nodes roughly equally between the two zones, in half of our realizations, the nodes with k equal to the limit belong to the internal zone, and in the other half, they belong to the external zone. We will then show results of simulations for ER networks with <k>=20 and *N* = 10,000, at the point of breakdown κ=2, where $\kappa =\frac{<k^{2}>}{<k>}$.

## 4. k-Shell Model

This is the model built by the k-shell decomposition method. Initially, following the procedure explained in [25] we decompose the network into k shells. We recursively remove nodes with degree k or less, by increasing k, starting with k=1. Each k-shell includes the nodes that were removed when all nodes with degree k were removed. This procedure is continued for *k* = 2, 3 etc., for all k values, and it stops when all nodes are removed. We divide the network nodes into 2 zones according to their k-shell. We assume that the nodes of the nucleus, i.e., the nodes of the last k-shell, will belong to the internal zone while the nodes of all the other k shells will belong to the external zone. The network breaks down when κ reaches the limiting value of κ=2.

The implemented python codes for both models are freely available in an open access repository [26].

## 5. Results

**Figure 2:**
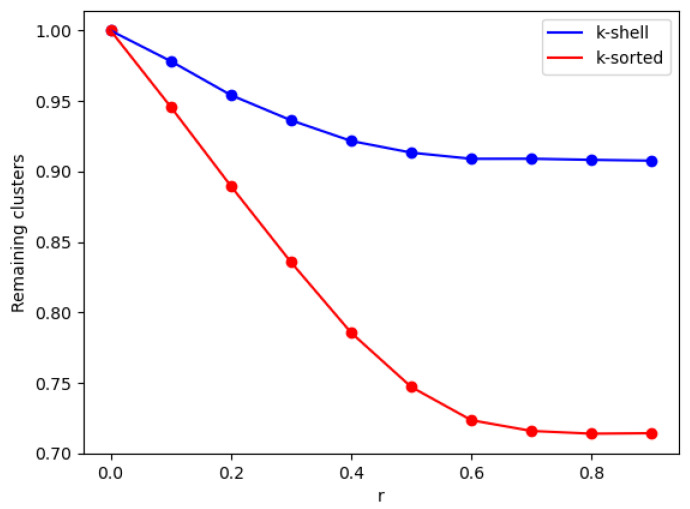
Normalized number of clusters (The normalization has been calculated at the maximum value at the critical point at r=0) as a function of *r*, on ER (Erdős–Rényi) networks with <k>=20 and *N* = 10,000, for the k-shell (blue, top line) and k-sorted model (red, bottom line) at the network breakdown point (κ = 2). The solid lines are optical guides. The ranges of error bars in y-axis are between $8.2\times 10^{-4}$ and $8.9\times 10^{-4}$ and $7.6\times 10^{-4}$–$8.9\times 10^{-4}$ for the k-shell and k-sorted model, respectively, for all *r* values.

We implement the two models that we discussed above using regular Erdős–Rényi networks. For each model, we divide the network into two zones, an inner zone and an outer zone. We then start to take out nodes (and their connected links) randomly, with two different probabilities for the inner and outer zone. We stop when a spanning cluster ceases to exist, and we monitor several properties of the resulting network. In Figure 2, we plot the number of clusters that have remained at the network, as a function of r, for both models at the critical point of the network breakdown, i.e., when κ=2. We observe that the higher the difference of the removal probabilities between the two zones is, the smaller is the number of generated clusters. This observation applies to both models that we investigated, albeit with a considerable difference between the two. We observe that with the k-shell model fewer nodes need to be removed so as to achieve network breakdown, which signifies the importance of considering the degree of the nearest neighbor, as well as the degree of the neighbor of the nearest neighbor as an impactful factor.

**Figure 3:**
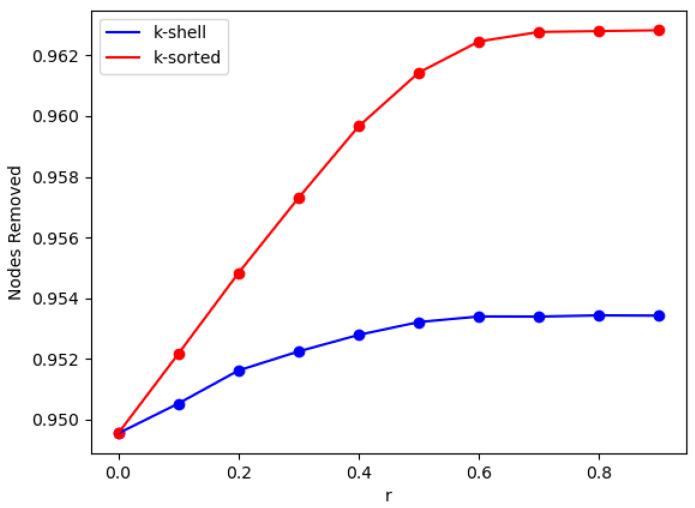
Number of removed nodes (as a fraction of the total nodes) as a function of *r*, derived by simulations of the k-shell (blue, bottom line) and the k-sorted model (red, top line), on ER networks with <k>=20 and *N* = 10,000, at the network breakdown point (κ=2). The solid lines are optical guides. The ranges of error bars in y-axis are between $5.78\times 10^{-5}$ and $6.24\times 10^{-5}$ and $5.29\times 10^{-5}$–$6.24\times 10^{-5}$ for the k-shell and k-sorted model, respectively, for all *r* values.

In Figure 3, we monitor the number of nodes that have to be removed so that the critical point is reached. We observe here that the higher the difference of the removal probabilities between the two zones is, the larger is the number of removed nodes needed so as to reach the critical point.As seen from Figure 2 and Figure 3, increasing the parameter *r* above 0.5 does not improve the break-up of the network by much. This could guide the radiation therapist’s dose increases applied to a given tumor, when combined with repeated CT imaging of the tumor cell network, e.g., when combined with a tumor cell targeted contrast element [27].

**Figure 4:**
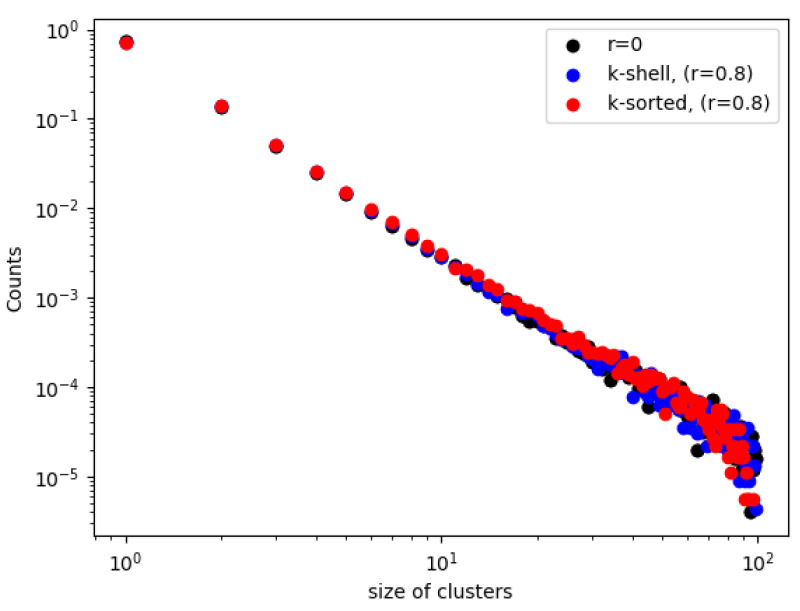
Distributions of the sizes of clusters for the two models at the critical point κ=2 in log–log plot for r=0 and r=0.8. For r=0 the distributions are identical for the 2 models (black dots). For r=0.8, the distributions for the k-shell model (red dots) and k-sorted model (blue dots) are not identical.

The distribution of the sizes of the clusters of Figure 2 are plotted in Figure 4 (for r=0 and for r=0.8). We observe in Figure 4, when the removal probabilities are the same for both zones, that the distributions are identical (black dots). When the inner zone has a smaller probability than the outer zone, then there is just a small difference, where there are slightly more small sized clusters in the k-shell than in the k-sorted model, which agrees with the results of Figure 2. However, this difference is within statistical error. However, it is interesting to see that there exists a very good linear relationships in the log–log plots, which means that all these distributions follow a nice power law form, for both models. We calculate the slope of the straight lines in Figure 3 to be −2.37 (black), −2.29 (blue) and −2.34 (red). Surprisingly, within statistical error, the slopes of the cluster size distribution for the two cases, with zones of different probabilities, and without any zones, are similar when the network breaks down. This implies that depleting zones with different probabilities plays no role in the fragments that are broken off the main body of the network at the critical point exactly when a spanning cluster ceases to exist.

**Figure 5:**
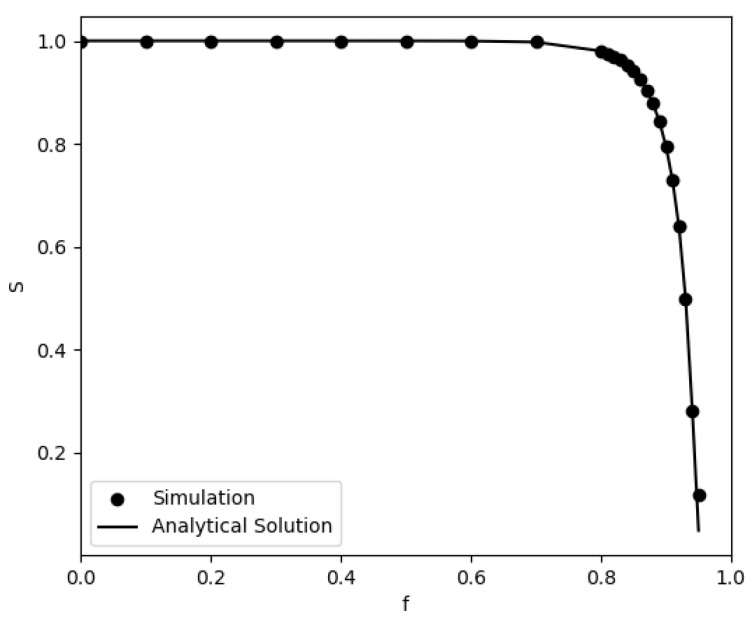
Analytical (solid line) and simulation (dots) results for the relative size of the largest cluster *S* during the entire removal process for the k-shell model as a function of the fraction of removed nodes for r=0. The networks have size *N* = 10,000 and <k>=20.

For the case of a network breakdown with only one zone (i.e., equal probabilities of node removal in the entire network), there is an analytical solution for the relative size of the largest cluster *S*, as a function of the number of removed nodes [28] where *S* is defined as the fraction of the nodes that belong to the largest cluster to the remaining nodes in the network. In Figure 5, we plot this analytical solution together with our simulation results only for the case of r=0. To derive the analytical solution of *S*, we calculate the equation

(1) $$S=1-G_{0}\left(u\right)$$

where

(2) $$G_{0}\left(x\right)=\sum_{k=0}^{\infty }P\left(k\right)x^{k}$$

(3) $$G_{1}\left(x\right)=\frac{1}{<k>}G_{0}^{\prime }\left(x\right)$$

*u* is the smallest non negative solution of the equation $u=G_{1}\left(u\right)$ and P(k) is the degree distribution of the network. We observe excellent agreement between the simulations and the analytical solution. Notice that the normalization on the *y*-axis is done by dividing the size of the largest cluster by the total number of nodes that are present at any given value of *f*. If the normalization is done by dividing by *N*, the initial total number of nodes, then the curve is almost linear, in agreement with Albert-Barabási [28].

**Figure 6:**
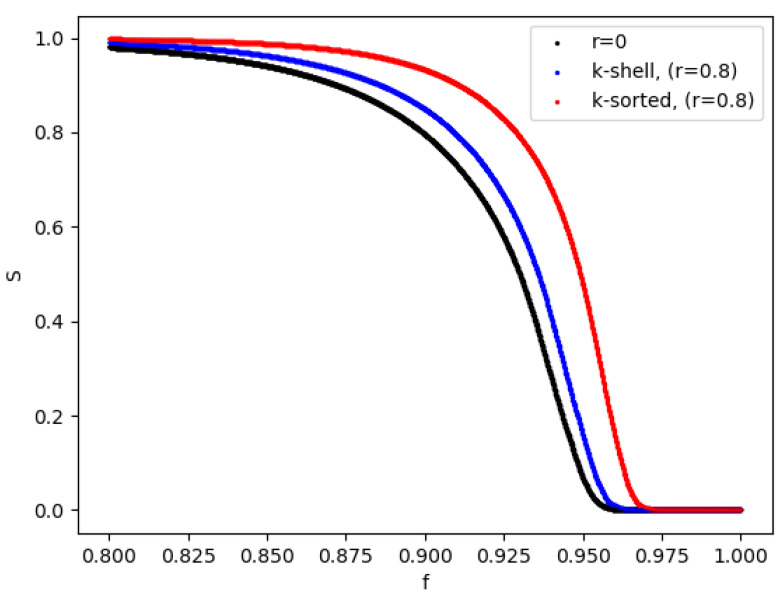
Relative size of the largest cluster *S* during the entire removal process for the two models, for r=0 and r=0.8, as a fraction of the removed nodes. For r=0 the values of *S* for the two models do overlap (black, bottom line). For r=0.8 we observe 2 distinct curves for the k-shell (blue, middle line ) and the k-sorted model (red, top line). The networks have size N=10,000 and <k>=20.

In order to have an indication for how the network changes during the entire removal process, we show in Figure 6 the change in the size of the largest cluster as a function of the fraction of the removed nodes, which, in essence, is as a function of time. When the removal probabilities are identical in the two zones, then we have identical results (black line). When the inner zone has a smaller removal probability than the outer zone, we observe that the largest cluster is reducing in size faster for the k-shell model (blue) than for the k-sorted model (red).

Considering Figure 6, we clearly see that by reducing the node removal probability in the inner zones (red and blue lines), the size of the remaining network (“largest cluster”) increases significantly. Say at 95% removal (9500 nodes removed, f=0.95), compared to the black line (r=0), the k-shell model of removal (blue line) gives a roughly 3 times larger “largest cluster” *S* (the remaining intact network), while the alternative model (red line) gives an about 7 times larger “largest cluster” *S*. This is a manifestation of the fact that in the k-shell model about 85% of the network nodes belong to the inner zone, while in the k-sorted model almost exactly half of the nodes do. This is so because in the k-shell model the number of nodes in the inner zone comes out automatically as the denser part of the network, while in the k-sorted model we define the cutoff line as the median in the list of the sorted nodes according to their k value.

Finally, we also implemented some other algorithms in an effort to properly divide the network nodes into zones. First, we randomly chose half of the nodes to belong to the inner zone and the other half to the outer zone. We then applied the algorithm with reduced probability of removal in the inner zone, as compared to the outer zone. The results for the properties monitored earlier, i.e., the number of clusters, the number of nodes removed, the size of the largest spanning cluster, etc., did not follow any particular trend but just produce random noise, meaning that the random division of nodes into zones does not properly identify the system’s core.

Next, we tried an embedding algorithm so as to cast the network on a two-dimensional surface, which would give measurable geometrical distances (x and y values) between the nodes. We did this by using two algorithms, a force directed algorithm, and the node2vec embedding algorithm [29]. The results did not follow a specific pattern, just as in the above random zones approach. Again, we conclude that these are not proper ways for identifying the core of the network.

## 6. Discussion and Conclusions

Qualitatively, the results of these network models resemble those of the lattice model [1]: A drop in the node removal (cancer kill) probability between the tumor’s external and internal zones decelerates the network’s breakdown. Quantitatively, at about 95% site (cell) destruction, we here get an order of magnitude increase in the size of the surviving network (see Results section). Medically this would be bad news, of course. However, it is unlikely that the radiation beam hitting the inner zone of the tumor (its center) would be weaker than that hitting the outer zone (periphery). On the other hand, the chemical factor that reduces the radiotherapy efficacy, hypoxia, is more significant at the tumor’s center, while at its periphery the oxygen level is expected to be that of normal tissue, where there is no hypoxia, by definition [10]. Similarly, the therapy resistance causing acidosis and hyperkalemia are more severe in the tumor’s central zone [2,5,6,7,8,9,10], where acidosis is going to reduce the efficacy of both chemotherapy and radiochemotherapy. Potentially, remedying such radiotherapy failures as demonstrated here may involve intensifying the radiation beam center, i.e., hitting the tumor’s center harder; however, technical and medical challenges will have to be addressed. On the other hand, ways may be found for reducing the hypoxia in the tumor’s center using micro- or nano-technology.

In conclusion, this new mathematical network breakdown model may illuminate present therapy challenges, due to tumor hypoxia and acidosis, and inspire future solutions regarding cancer radiotherapy and radiochemotherapy. In addition, due to the increased hyperkalemia in the tumor’s inner zone, this 2-zone model may as well be relevant to immunotherapy. Having shown the importance of a reduced site elimination probability in a network’s inner zone underlines the importance of chemical imaging of a tumor’s inner zone heterogeneities, whether in a patient or a patient’s xenograft.

Finally, we point out that chemical images of real tumors, in vivo, together with tumor therapy efficacy maps, are being derived in one of our laboratories. The derived tumor maps could serve as the matrix for future analysis, as has been done here, regarding the *r* parameter, so as to help optimize the spatial contours of the radiation beam.

## Data Availability

The data presented in this study are openly available in an open access repository [26].
